# Impacts of national volume-based drug procurement policy on the utilization and costs of antihypertensive drugs in a Chinese medicine hospital: an interrupted time series analysis of 5138 patients

**DOI:** 10.3389/fphar.2024.1302154

**Published:** 2024-02-08

**Authors:** Lili Shang, Yan Cheng, Jifang Zhou, Yuqing Bao, Desong Kong, Ruijian Huang, Yanfei Chen, Hao Wang, Ning Gu, Aixia Ma

**Affiliations:** ^1^ School of International Pharmaceutical Business, China Pharmaceutical University, Nanjing, China; ^2^ Department of Discipline Construction, Nanjing University of Chinese Medicine, Nanjing, China; ^3^ Department of Pharmacy, Nanjing Hospital of Chinese Medicine Affiliated to Nanjing University of Chinese Medicine, Nanjing, China; ^4^ Chinese Medicine Modernization and Big Data Research Center, Nanjing Hospital of Chinese Medicine Affiliated to Nanjing University of Chinese Medicine, Nanjing, China; ^5^ Department of Pharmacy, Nanjing Drum Tower Hospital, The Affiliated Hospital of Nanjing University Medical School, Nanjing, China; ^6^ Cardiovascular Department, Nanjing Hospital of Chinese Medicine, Nanjing, China

**Keywords:** antihypertensive drugs, healthcare expenditures, interrupted time series, national centralized drug procurement, volume-based procurement antihypertensive drugs, national volume-based drug procurement

## Abstract

**Objectives:** The study aimed to estimate the effects of National Volume-based Drug Procurement (NVBP) policy on drug utilization and medical expenditures of hypertension patients in public medical institutions in mainland China.

**Methods:** This study used patient-level data based on electronic health records retrieved from the hospital information system of Nanjing Hospital of Chinese Medicine. Data on patients with hypertension who received care at this institution between 2016 and 2021 was used for analysis. Segmented linear regression models incorporating Interrupted Time Series (ITS) analysis were adopted to examine the effects of NVBP policy on drug utilization and health expenditures of eligible patients. Drug utilization volume and health expenditures were the primary outcomes used to assess the policy effects, and were measured using the prescription proportion of each drug class and the overall per-encounter treatment costs.

**Results:** After the implementation of NVBP policy, the volume of non-winning drugs decreased from 54.42% to 36.25% for outpatient care and from 35.62% to 15.65% for inpatient care. The ITS analysis showed that the volume of bid-winning drugs in outpatient and inpatient settings increased by 9.55% (*p* < 0.001) and 6.31% (*p* < 0.001), respectively. The volume changes in non-volume based purchased (non-VBP) drugs differed between outpatients and inpatients. The proportion of non-VBP drugs immediately increased by 5.34% (*p* = 0.002) overall, and showed an upward trend in the outpatient setting specially (*p* < 0.001) during the post-intervention period. However, no significant differences were observed in the proportion of non-VBP drugs in inpatient setting (*p* > 0.05) in term of level change (*p* > 0.05) or trend change (*p* > 0.05). The average per-visit expenditures of outpatients across all drug groups exhibited an upward trend (*p* < 0.05) post policy intervention. In addition, a similar increase in the overall costs for chemical drugs were observed in inpatient settings (coefficient = 2,599.54, *p* = 0.036), with no statistically significant differences in the regression slope and level (*p* = 0.814).

**Conclusion:** The usage proportion of bid-winning drugs increased significantly post policy intervention, indicating greater use of bid-winning drugs and the corresponding substitution of non-winning hypertensive drugs. Drug expenditures for outpatients and health expenditures per visit for inpatients also exhibited an upward trend, suggesting the importance of enhanced drug use management in Traditional Chinese Medicine hospital settings.

## 1 Introduction

China has been actively controlling the rapidly growing medical costs and alleviating the financial burdens of medical care for patients. However, drug expenditures continue to increase year on year (Tao et al., 2020). According to China Health Statistical Yearbook 2021, China’ total health expenditure has grown rapidly from 458.4 billion Chinese Yuan (CNY) in 2000–7,217.5 billion CNY in 2020 in China (Ma et al., 2021). Over the past 20 years, this total has grown by 15.7 times the starting amount, with an annual growth rate being higher than GDP growth rate per year. Although the proportion of drug expenditure has declined in recent years, total drug expenditures were 223.8 billion CNY in China over this period, accounting for 31.0% of overall health expenditure, which is far higher than the average level of 17% in the Organization for Economic Cooperation and Development (OECD) countries (Papanicolas et al., 2018; Wang et al., 2021a). Drug expenditures are subject to both unit drug prices and drug utilization volume, which correspond to the pharmacoeconomic supply and demand sides, respectively (Han et al., 2015). Thus, preventing rising drug prices is a much-noted potential solution to reducing medical expenditures (Wen et al., 2021).

To ensure a patient’s choice of treatment options while mitigating price hikes on essential medications for chronic conditions, Chinese government issued a National Centralized Drug Procurement (NVBP) policy in January 2019 (The people’s Republic of China, 2019). The NVBP policy represents the first attempt at nationwide volume-based drug procurement in mainland China, aiming to provide patients with high-quality drugs at lower prices through economies of scale. Four municipalities and seven sub-provincial cities in mainland China were selected as pilot cities for the first round of the NVBP policy’s implementation, with this pilot policy introduction subsequently becoming known as the “4 + 7” policy (Li and Bai, 2019).

To date, seven rounds of NVBP pilots have been conducted and implemented in all tertiary level-A hospitals nationwide. The current drug list for NVBP pilots has expanded and continued to be implemented in more regions. Unlike provincial-based drug procurement pilots, NVBP policy is organized by the central government’s procurement division. Under supervision of the State Council, the Joint Procurement Office (JPO) was established as an alliance formed by representatives of local drug procurement agencies. Thus, NVBP policy received an unprecedented level of political commitment (Yuan et al., 2021).

The primary strength of the NVBP policy lay in its “volume-based procurement”, which encompassed several unique policy measures. First, volume-price linkage of procured drugs was achieved (Qu et al., 2021). Pharmaceutical enterprises offered maximum discounts on drug prices to obtain a larger market. Second, all generic drugs became obligated to pass a Generic Consistency Evaluation (GCE) before being listed for procurement. This ensured that generic drugs were adequately screened for consistency in quality and efficacy in line with those of their corresponding brand name drug equivalents (The people’s Republic of China, 2016). Third, the NVBP policy guaranteed utilization of bid-winning drugs, improved the drug supply chain efficiency, and reduced capital and marketing costs (Yuan et al., 2021).

Previous studies have reported that after the implementation of the NVBP policy, non-winning generic drugs were substituted with bid-winning original and generic drugs (Lu et al., 2022a). The daily cost of bid-winning original and generic drugs decreased significantly, as did that of non-winning drugs (Yang et al., 2022). After the NVBP policy, the utilization of generic cardiovascular drugs was increasing, and the drug expenditure had been reduced by 61% (Wang et al., 2020a). A national survey revealed that average medication affordability improved from 8.2 days’ wages to 2.8 days’ wages, and that all the bid-winning cardiovascular drugs became affordable following the NVBP policy (Yuan et al., 2021). Relevant studies have also reported that NVBP policy played a positive role in reducing the overall drug costs and improving the average medication affordability (Li et al., 2019; Chen et al., 2020; Zhang et al., 2022). However, some researchers indicated that while total medical expenditures and drug costs decreased by 11.4% and 13.8%, respectively, other expenditures increased by 16.3% (Li et al., 2021). A similar finding was found that drug expenditures were effectively reduced while, overall health expenditures increased over time (Shen et al., 2021). In other words, Price cuts alone may be insufficient to reduce the overall healthcare burdens of patients (Kwon et al., 2019). Other studies have clarified that price reduction alone did not necessarily lead to a decrease in overall healthcare economic burden (Zhen et al., 2018; Yang et al., 2021a).

Many existing research conclusions primarily rely on drug purchase data from regional or institutional sources of comprehensive hospitals, rather than more detailed individual prescription data. This could limit the generalizability of findings concerning the effects of policies. Furthermore, several aspects of the NVBP policy’s impact remain ambiguous. For instance, drug usage and healthcare costs could vary in different pilot cities. Mainly because the baseline drug use structure varied in cities and insufficient payment capacity of local health insurance funds. Similarly, the policy effectiveness might vary in different healthcare environments. In China, healthcare facilities range from comprehensive hospitals emphasizing Western medicine to Traditional Chinese Medicine (TCM) hospitals. Unlike Western-oriented hospitals, TCM hospitals offer patients both traditional Chinese medicinal treatments and non-pharmacological TCM therapies like acupuncture and massage. The zero-markup policy has varied effects across these healthcare settings. Specifically, the impact of the policy appears more pronounced in Western medicine hospitals compared to TCM hospitals (Jiang et al., 2020). Due to the above reasons and further in depth analysis by using real-world data, we conducted a quantitative study examining the shifts in the use of hypertensive medications and the medical expenses of patients. The selection of hypertensive patient was primarily based on the fact that centralized procurement involved many category of antihypertensive drugs on the list. Another reason was that a high prevalence of hypertension, which made it easier to obtain sufficient number of research subjects. So, this analysis was based on individual patient data from a TCM hospital, comparing periods before and after the introduction of the NVBP policy.

## 2 Materials and methods

### 2.1 Data sources and collection

Real-world prescription and expenditure data for hypertension patients was sourced from the Hospital Information System (HIS) of a Chinese Medicine Hospital in Nanjing. Nanjing, the capital city of Jiangsu Province, is a megacity in South China. Nanjing consists of 11 districts, with a total administrative area of 6,587.04 km^2^ and a total population of 9.49 million in 2022. By the end of 2021, Nanjing has 3,451 medical institutions, of which 277 are hospitals. In Nanjing, the overall clinical visits are 84.72 million in 2021, the medical costs per time per patient in outpatient and inpatient are 360.78 CNY and 17,550.41CNY, respectively. This Chinese Medicine Hospital is a prominent tertiary-grade level-A Chinese Medicine hospital, housing about 1,500 regular beds. The study targeted patients diagnosed with hypertension who had medication records spanning from 1 January 2016, to 30 April 2021.

Exclusion criteria were set to filter out specific patients.

1. Those with secondary or refractory hypertension, hypertension accompanied by valvar heart disease, or hypertension coexisting with severe, debilitating chronic illnesses like cancer or liver diseases.
2. Primary hypertension patients missing essential demographic or clinical details.

The extracted data covered a wide range, encompassing diagnostic details, demographic information, patient visit logs, prescription records, hospitalization details, imaging, consultations, lab tests, and expenditure. Specifically, the medication data incorporated drug names, specifications, dosage forms, quantities purchased, and manufacturing details.

Till 30 April 2021, the four rounds of NVBP have been implemented. To gauge the impact of the NVBP policy on various hypertension medications, drugs derived from HIS were segmented into NVBP policy-related drugs and non-volume based purchased (non-VBP) drugs. The NVBP policy-related drugs were sorted into bid-winning drugs and non-winning drugs based on the bidding results of four rounds of NVBP, otherwise they were deemed to non-winning drugs. Specific timing had been set for the bid-winning drugs involved in the four rounds of NVBP within the research period. The antihypertensive drugs, which were not included in the list of the four batches of NVBP, were defined as non-VBP drugs. Expenditure data encapsulated total healthcare costs, drug-related costs, consultation fees, and other expenses. This “other” category consisted of services, materials, treatment operations, and surgeries. Furthermore, participants were bifurcated based on whether they were outpatients or inpatients.

Adopting a retrospective methodology, the study took on the lens of a healthcare provider when analyzing costs. The study secured approval from the Research Ethics Committee of Nanjing Hospital of Chinese Medicine, under the code KY2021091. Electronic health records (EHR) data from HIS has recently received increasing recognition as a rich real-world research resource for studying healthcare issues (Huser and cimino, 2013; Chalmers et al., 2016; Cowie et al., 2017). EHR data were subsequently used in this study to gain a more well-rounded understanding of the real-world drug utilization and medical expenditures of patients with hypertension before and after the implementation of the NVBP policy.

### 2.2 Outcome variable

This study evaluated the impact of NVBP policy on drug utilization and expenditures of hypertension patients. So, the primary outcomes were the drug use volume and expenditures. The drug use volume was measured by the prescription proportion of drugs, which was calculated as the total number of prescriptions with the same category as a proportion of the summed prescriptions for enrolled hypertension patients in the month. To evaluate the effect on policy-related drugs, the chemical antihypertensive drugs were divided into three categories: winning, non-winning drugs, and non-VBP drugs. The prescription proportion was opted to be used because the aim was not only to observe changes in the volume at patient-level, but also to examine alterations in medication structure of outpatients and inpatients during the observation period. Expenditure data was measured by CNY. The average health expenditure per visit was calculated as the overall healthcare expenditures in accordance with the number of patient visits in a given month. The average monthly drug expenditures, consultation expenditures and other expenditures were calculated in the same manner. Hypertension patients’ average monthly health expenditures were calculated using the following formula:

Whereas b_i_ represents the specific kind of expenditure incurred by outpatients/inpatients each time, n represents the total numbers of outpatient/inpatient visits in a given month, and N represents total number of outpatients/inpatients in the month.

### 2.3 Statistical analysis

Descriptive statistics were used to quantify the change in drug use volume of each category and expenditures of hypertension patients before and after the implementation of NVBP policy. Graphical displays of the use volume of each drug category and the expenditure variables were created to visualize the monthly changes in drug use volume and patient burden over the period covering from January 2016 to April 2021.

A single-group interrupted time series (ITS) was designed to assess the policy effects on drug use volume and expenditures of patients diagnosed with hypertension. ITS is a commonly used approach to evaluate the longitudinal effects of interventions that occur at a fixed point in time (Wagner et al., 2002). The time unit was set to 1 month and the intervention time was set to January 2020, which time was the policy implemented in the hospital. Therefore, a monthly time series was constructed involving 64 time points between January 2016 and April 2021, including 48 points pre-intervention and 16 points post-intervention. Owing to the lack of data available prior to 2019, the monthly time series of outpatients was obtained at 28 different points between January 2019 and April 2021, including 12 pre-intervention and 16 post-intervention points. Segmented regression models were used that controlled for baseline trends to estimate changes in the levels and trends of each outcome variable after the implementation of the NVBP policy. The following segmented linear regression model was developed:

Y_t_ refers to the outcome variable (volume or expenditures) in month *t*, and time is a continuous variable indicating time in months at time t from the start of the observation period; intervention is an indicator for time t occurring before (intervention = 0) and after (intervention = 1) NVBP policy; time after intervention indicates months passed since the intervention (time prior to the intervention is coded 0). In this model, *ß*_0_ represents the baseline level of outcome variable at the beginning of the observation period. *ß*_1_ estimates the linear trend during the pre-intervention period. *ß*_2_ estimates the change in level immediately following policy intervention. β3 estimates the differences between pre- and post-intervention slopes. ε_t_ is an estimate of the random error at time t. Durbin–Watson test was performed to test the presence of first-order auto-correlation, a Durbin–Watson value obtained of around 2 indicates no sign of auto-correlation. R version 4.2.1 was used to perform the ITS analysis.

A sensitivity analysis was carried out to validate the robustness of the results. During the observation period, four rounds of VBP batches had been implenmented, we appropriately specified the implementation time of different rounds during the policy promotion period to 1 January 2020, which was the first implementation time of NVBP policy in the hospital, and conducted ITS analysis. Furthermore, the absolute prescription volume was chosen an the indicator to investigate the volume change after policy intervention.

## 3 Results

### 3.1 Descriptive analysis

**FIGURE 1:**
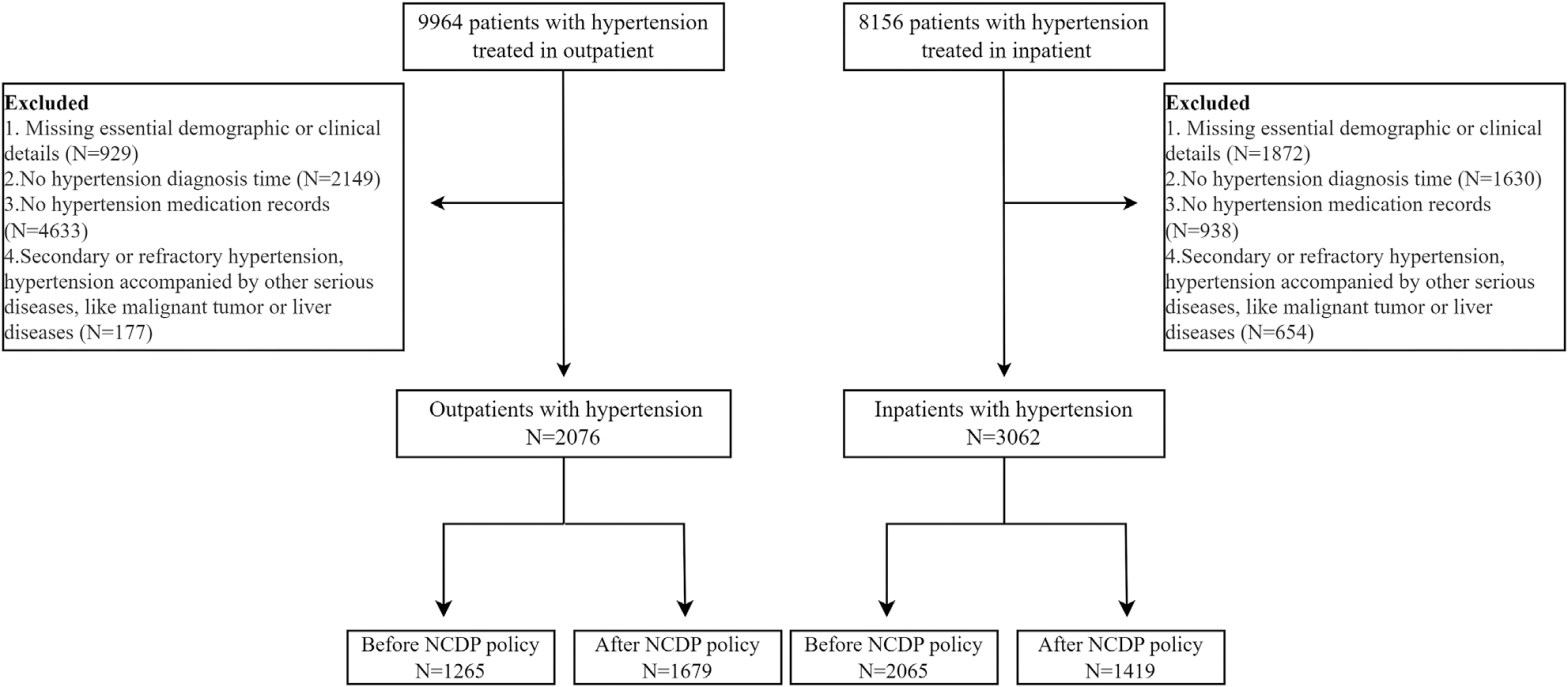
Patients enrolment into the study.

5,138 hypertension patients covering the period from January 2016 to April 2021, met the relevant inclusion criteria, and were subsequently included in this study, involving 2076 outpatients and 3,062 inpatients. The inclusion and exclusion criteria were shown in Figure 1. As shown in Table 1, no significant difference in socio-demographic characteristics of hypertension patients enrolled the research was observed before and after the policy, whether in the outpatients or inpatients settings.

**TABLE 1:** Clinical characteristics of outpatients and inpatients before and after the implementation of NVBP policy.

Characteristics	Outpatients			Inpatients		
	Before (n = 1,265)	After (n = 1,679)		Before (n = 2,065)	After (n = 1,419)	
	n (%)	n (%)	*p*	n (%)	n (%)	*p*
Male	659 (52.1)	880 (52.4)	0.865	1,027 (49.7)	731 (51.5)	0.301
Age(y)			0.876			0.423
<60	135 (10.7)	181 (10.8)		19 (0.9)	15 (1.6)	
60∼	687 (54.3)	949 (56.5)		646 (31.3)	431 (30.4)	
70∼	213 (16.8)	285 (17.0)		713 (34.5)	463 (32.6)	
≥80	230 (18.2)	264 (15.7)		687 (33.3)	510 (35.9)	
Insurance			0.593			0.005
UEBMI	693 (54.8)	902 (53.7)		1,696 (82.1)	1,217 (85.8)	
Other insurance	572 (45.2)	777 (46.3)		369 (17.9)	202 (14.2)	
Comorbidity						
MI	7 (0.6)	10 (0.6)	0.881	26 (1.3)	22 (1.6)	0.469
Apoplexy	2 (0.2)	1 (0.1)	0.407	4 (0.2)	13 (0.9)	0.003
Dyslipidemia	101 (8.0)	136 (8.1)	0.909	83 (4.0)	86 (6.1)	0.006
Diabetes mellitus	394 (31.1)	519 (30.9)	0.891	793 (38.4)	567 (40.0)	0.355
CKD	121 (9.6)	149 (8.9)	0.520	210 (10.2)	92 (6.5)	<0.001
Class of antihypertensive drugs						
CCB	820 (64.9)	1,019 (60.7)	0.022	1,427 (69.1)	989 (69.7)	0.709
ACEI	130 (10.3)	161 (9.6)	0.536	386 (18.7)	252 (17.8)	0.484
ARB	490 (38.8)	581 (34.6)	0.021	720 (34.9)	466 (32.8)	0.215
Diuretic	175 (13.8)	233 (13.9)	0.973	551 (26.7)	474 (33.4)	<0.001
β-blocker	399 (31.5)	492 (29.3)	0.191	584 (28.3)	473 (33.3)	0.001
α-blocker	23 (1.8)	29 (1.7)	0.853	122 (5.9)	129 (9.1)	<0.001
Compound preparation						
ARB + Diuretic	328 (25.9)	381 (22.7)	0.042	382 (18.5)	209 (14.7)	0.004
ARB + CCB	302 (23.9)	356 (21.2)	0.085	317 (15.4)	199 (14.0)	0.279
ARB + other drug	13 (1.0)	27 (1.6)	0.178	19 (0.1)	28 (2.0)	0.008
ACEI + Diuretic	88 (7.0)	93 (5.6)	0.113	43 (2.1)	10 (0.7)	0.001
ACEI + CCB	63 (5.0)	120 (7.1)	0.016	24 (1.2)	50 (3.5)	<0.001
TCM	731 (57.8)	933 (55.6)	0.023	1823 (88.3)	1,216 (85.7)	0.025

Abbreviations: UEBMI, urban employee basic medical insurance; MI, miocardial infarction; CKD, chronic kidney disease; CCB, calcium channel blocker; ACEI, angiotensin converting enzyme inhibitor; ARB, angiotensin receptor blocker.

**FIGURE 2:**
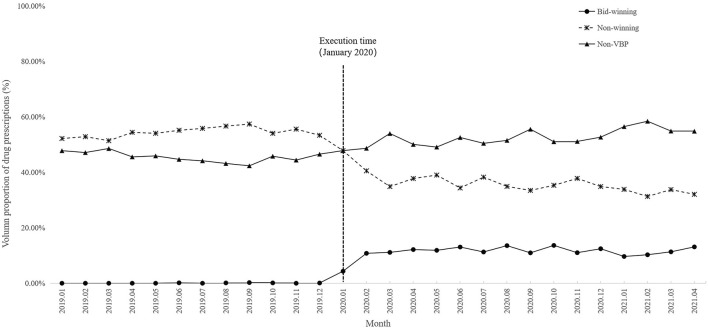
Trends in monthly volume proportion of bid-winning, non-winning and non-VBP antihypertensive drugs in outpatient setting from January 2019 to April 2021.

**FIGURE 3:**
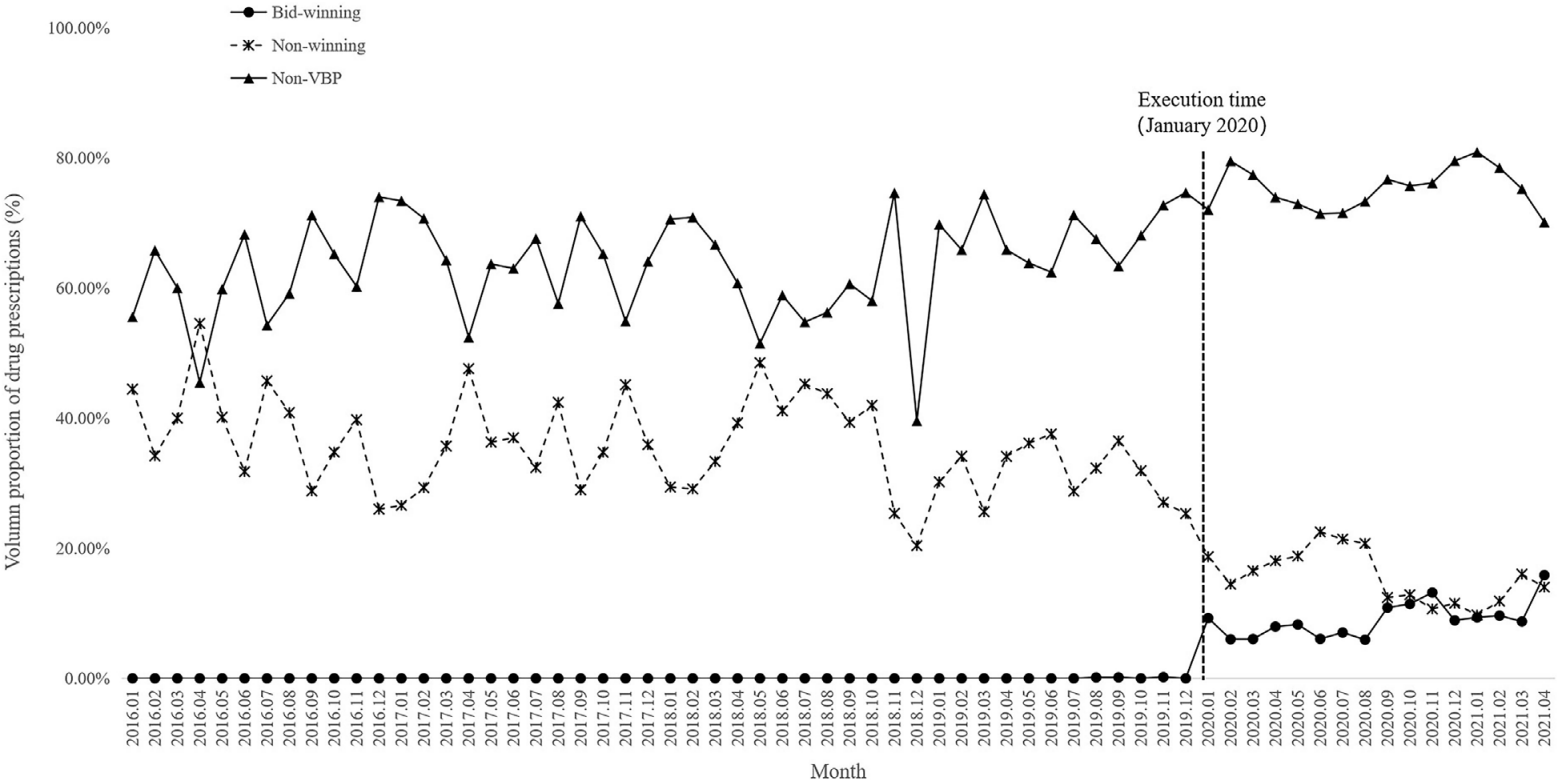
Trends in monthly volume proportion of bid-winning, non-winning and non-VBP antihypertensive drugs in inpatient setting from January 2016 to April 2021.

A total of 32 antihypertensive drugs related to the NVBP policy between January 2020 and April 2021 were included in this study. Among them, 11 were bid-winning drugs and 21 were non-winning drugs (Supplementary Table S1). Monthly trend charts of the use proportion of bid-winning, non-winning and non-VBP drugs among outpatients and inpatients are shown in Figure 2 and Figure 3. The proportion of bid-winning antihypertensive drugs increased remarkably, while the non-winning drugs prominently decreased after the implementation of NVBP policy. As shown in Table 2, the volume proportion of bid-winning drugs increased by 11.29% for outpatients and rose from 0.01% to 9.04% for inpatients before intervention period and after intervention period, whereas downward trends were observed for non-winning drugs. The volume proportion of non-VBP drugs increased by 15.24% in outpatients and 18.53% in inpatients after the policy implementation, respectively.

**TABLE 2:** Changes of volume proportion of bid-winning, non-winning and non-VBP drugs before and after implementation of NVBP policy.

Categories	Outpatient department			Inpatient department		
	Before	After	Growth rate (%)	Before	After	Growth rate (%)
Bid-winning drugs	0.06	11.29	18,579.41	0.01	9.04	90,340.71
Non-winning drugs	54.42	36.25	−33.38	35.62	15.65	−56.07
Non-VBP drugs	45.52	52.46	15.24	63.54	75.31	18.53

**FIGURE 4:**
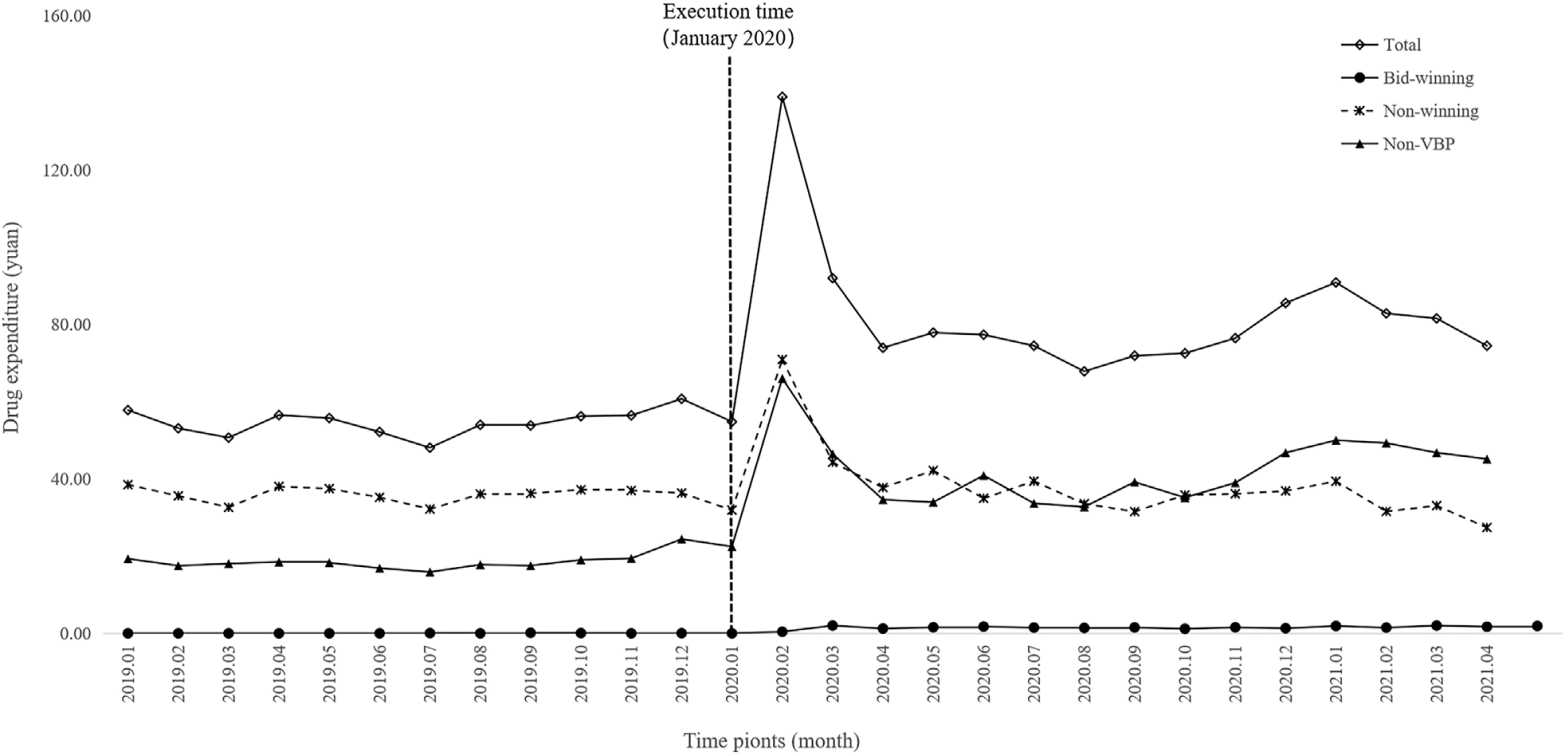
Trends in monthly drug expenditures of outpatients per visit from January 2019 to April 2021.

**FIGURE 5:**
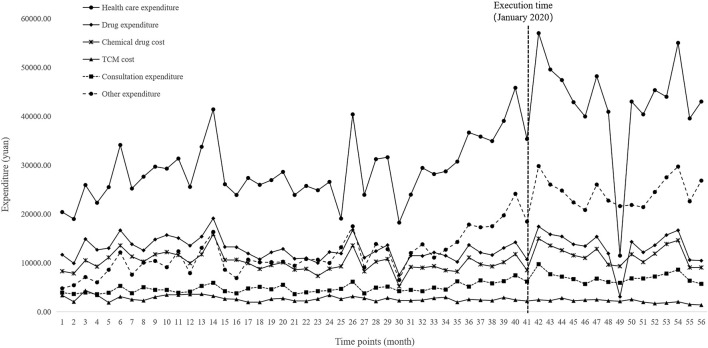
Trends in monthly healthcare expenditures of inpatients per visit from January 2016 to April 2021.

Figure 4 and Figure 5 visualized trends in monthly expenditures among outpatients and inpatients per visit before and after the implementation of NVBP policy, the drug expenditures of outpatients with hypertension increased, as well as the health expenditures in inpatient setting. As shown in Table 3, after policy implementation, the average expenditures for total drugs, non-winning drugs and non-VBP drugs per visit increased by 48.03%, 5.23% and 123.10%, respectively, in the outpatient setting. The expenditure on bid-winning drugs was only 1.53 yuan for outpatients with hypertension. Overall healthcare, all drugs, western medicines, consultations and other associated expenditures increased by 48.62%, 14.69%, 46.58% and 109.51%, respectively. The out-of-pocket expenditures and TCM expenditures decreased by 2.17% and 21.41% (see Table 4). The length of hospital stay increased from 15.58 days to 19.02 days for hypertension patients before and after implementation of the NVBP policy.

**TABLE 3:** Changes of expenditures of hypertension patients in outpatient setting before and after implementation of NVBP policy.

Categories	Before (yuan)	After (yuan)	Growth rate (%)
Total drugs	54.63	80.86	48.03
Bid-winning drugs	0.02	1.53	6,343.48
Non-winning drugs	36.05	37.93	5.23
Non-VBP drugs	18.56	41.40	123.10

**TABLE 4:** Changes of expenditures of hypertension patients in inpatient setting before and after implementation of NVBP policy.

Categories	Before	After	Growth rate (%)
Overall healthcare (yuan)	28,735.91	42,708.13	48.62
Drug expenditures (yuan)	12,800.87	13,166.63	2.86
Medical drug expenditures (yuan)	10,059.29	11,537.07	14.69
TCM expenditures (yuan)	2,741.58	2,154.47	−21.41
Consultation expenditures (yuan)	4,750.95	6,963.96	46.58
Other expenditures (yuan)	11,553.23	24,205.45	109.51
Out of pocket expenditures (yuan)	6,872.88	6,723.87	−2.17
Hospital days (day)	15.58	19.02	22.06

### 3.2 ITS analysis for the changes of volume

AS shown in Table 5. The prescription proportion of bid-winning drugs increased by 9.55 and 6.31 per month (*p* < 0.001) in the outpatient and inpatient settings after policy intervention, respectively. The trend change was not statistically significant (*p* = 0.459) among outpatients, whereas there was an upward trend (coefficient = 0.36, *p* < 0.001) among inpatients after intervention. A significant decline (−0.91 per month, 95%CI = −1.33 to −0.49, *p* < 0.001) was observed in the utilization of non-winning drugs among outpatients were found before and after implementation of the NVBP policy. The proportion of non-winning drugs decreased by 11.27% immediately post intervention (*p* = 0.013) in inpatient setting, though no statistically difference was found in the trend change (*p* = 0.459). For non-VBP drugs, the level and trend change showed an increase in terms of the prescription proportion for outpatients (*p* = 0.002 and *p* < 0.001, respectively), while no significant differences were observed in the volume changes (*p* > 0.05) for inpatients. The changes in TCM use were also analyzed post policy intervention. No significant changes were observed in the level or trend (both *p* > 0.05) of TCM in either outpatients or inpatients. There is no statistical difference in the volume use of TCM following the implementation of NVBP policy.

**TABLE 5:** ITS results of volume proportion of bid-winning, non-winning, non-VBP and TCM antihypertensive drugs.

Categories	Outpatients		Inpatients	
	Coef	95% CI	Coef	95% CI
Bid-winning				
Level change,β2	9.55	(6.87, 12.23)***	6.31	(5.10, 7.52)***
Trend change,β3	0.14	(-0.22, 0.49)	0.36	(0.24, 0.48)***
Durbin-Watson,d	1.86		1.76	
Non-winning				
Level change,β2	−15.84	(-19.19, −12.50)***	−11.27	(-19.84, −2.69)*
Trend change,β3	−0.91	(-1.33, −0.49)***	−0.33	(-1.20, 0.54)
Durbin-Watson,d	2.15		1.89	
Non-VBP				
Level change,β2	5.34	(2.41, 8.28)**	7.82	(-0.02, 15.66)
Trend change,β3	0.75	(0.38, 1.13)***	−0.01	(-0.78, 0.77)
Durbin-Watson,d	1.99		1.90	
TCM				
Level change,β2	0.19	(-32.08, 32.46)	−2.89	(-39.21, 33.44)
Trend change,β3	−2.75	(-7.70, 2.21)	−0.80	(-4.42, 2.82)
Durbin-Watson,d	1.94		1.97	

Abbreviations: Coef., coefficient; SE, standard error; CI, confidence interval.

*p < 0.05, **p < 0.01, ***p < 0.001.

### 3.3 ITS analysis for the changes of expenditures

The results of the ITS analysis for expenditure changes in outpatients with hypertension are shown in Table 6. The per visit total drug expenditures of outpatients increased by 31.11 yuan (*p* = 0.003). There were abrupt increases in expenditures for bid-winning drugs, non-winning drugs and non-VBP drugs. Expenditures on bid-winning products increased by 1.31 yuan (*p* < 0.001) after the intervention, following by an increasing trend (coefficient = 0.04, *p* = 0.049). The non-winning products increased by11.30 yuan (*p* = 0.010), but exhibited a downward trend (coefficient = −1.06, *p* = 0.046). Meanwhile, no significant change was observed for the trend of non-VBP drugs (*p* = 0.702). Among hypertension patients treated in inpatient setting, as shown in Table 7, the expenditures of total healthcare, overall western medicines, consultations and other associated expenditures immediately increased (*p* = 0.024, *p* = 0.036, *p* = 0.008 and *p* = 0.004, respectively) when NVBP policy was implemented. However, the slope changes in expenditures showed no statistically differences. Meanwhile, no significant changes were observed in the level (*p* = 0.119) or trend of all drug expenditures (*p* = 0.572). After the implementation of NVBP policy, the length of hospital stay showed an upward trend (coefficient = 2.37, *p* = 0.011), whereas the change trend before and after the implementation of NVBP policy was not apparent (coefficient = 0.04, *p* = 0.675).

**TABLE 6:** ITS results for drug expenditures in outpatient setting.

Categories	Coef	95% CI	*p*
Total drug expenditures			
Level change,β2	31.11	(12.45, 49.76)	0.003**
Trend change,β3	−0.74	(-3.07, 1.60)	0.541
Durbin-Watson,d	2.03		
Bid-winning drug expenditures			
Level change,β2	1.31	(1.03, 1.59)	<0.001***
Trend change,β3	0.04	(0.00, 0.07)	0.049*
Durbin-Watson,d	1.94		
Non-winning drug expenditures			
Level change,β2	11.30	(3.34, 19.27)	0.010*
Trend change,β3	−1.06	(-2.05, −0.07)	0.046*
Durbin-Watson,d	2.10		
Non-VBP drug expenditures			
Level change,β2	18.13	(7.02, 29.25)	0.004**
Trend change,β3	0.28	(-1.13, 1.68)	0.702
Durbin-Watson,d	2.00		

Abbreviations: Coef., coefficient; SE, standard error; CI, confidence interval.

*p < 0.05, **p < 0.01, ***p < 0.001.

**TABLE 7:** ITS results for healthcare expenditures in inpatient setting.

Categories	Coef	95% CI	*p*
Overall healthcare expenditures			
Level change,β2	10,140.00	(1,609.10, 18,670.90)	0.024*
Trend change,β3	−364.40	(-1,194.66, 465.86)	0.394
Durbin-Watson,d	2.00		
Drug expenditures			
Level change,β2	2,462.74	(-579.45, 5,504.93)	0.119
Trend change,β3	−86.03	(-382.50, 210.44)	0.572
Durbin-Watson,d	1.97		
Chemical drug cost			
Level change,β2	2,599.54	(236.25, 4,962.83)	0.036*
Trend change,β3	−27.87	(-258.62, 202.88)	0.814
Durbin-Watson,d	1.95		
TCM cost			
Level change,β2	210.68	(-364.93, 786.29)	0.476
Trend change,β3	−43.52	(-99.66, 12.62)	0.135
Durbin-Watson,d	1.98		
Consultation expenditures			
Level change,β2	1,490.40	(437.29, 2,543.51)	0.008**
Trend change,β3	−79.86	(-182.56, 22.84)	0.134
Durbin-Watson,d	2.00		
Other expenditures			
Level change,β2	5,753.79	(2012.48, 9,495.10)	0.004**
Trend change,β3	−72.51	(-438.01, 292.99)	0.700
Durbin-Watson,d	2.00		
Out of pocket expenditures			
Level change,β2	−45.78	(-2,696.37, 2,604.81)	0.973
Trend change,β3	145.86	(-115.11, 406.83)	0.278
Durbin-Watson,d	1.97		
Hospital days			
Level change,β2	2.37	(0.61, 4.14)	0.011*
Trend change,β3	0.04	(-0.13, 0.21)	0.675
Durbin-Watson,d	1.99		

Abbreviations: Coef., coefficient; SE, standard error; CI, confidence interval.

*p < 0.05, **p < 0.01, ***p < 0.001.

### 3.4 Sensitivity analysis

After the execution time of all bid-winning drugs was unified to 1 January 2020, which was the first implementation time of NVBP policy in the hospital. The volume propertion of bid-winning drugs increased and the proportion of non-winning drugs decreased both in the outpatient and inpatient settings (Supplementary Table S2). The changes of non-VBP were different in the outpatients and inpatients. The findings were consistent with those obtained using specific bidding times. In terms of absolute prescription volume, the ITS results showed that NVBP policy promoted the consumption of bid-winning antihypertensive drugs and suppressed the utilization of non-winning drugs. The immediate and long-term trends of non-VBP drugs were stable, with no change in the trends among inpatients before and after the NVBP policy implementation. However, the usage volume of non-VBP drugs increased after the policy implemented (Supplementary Table S3). The results were consistent with the findings using the prescription proportion of drugs. The sensitivity analysis proved the robustness of the research findings.

## 4 Discussion

To the best of the authors’ knowledge, this is the first study patient-level data in the real world have been used to evaluate the impact of NVBP policy on the associated drug use and expenditure relating to hypertension patients. Considering the differences in the treatment setting, we selected hypertension patients treated in outpatient and inpatient departments as the population for data collection to investigate the policy impact from different dimensions.

Overall, this study found that the NVBP policy promoted the consumption of bid-winning antihypertensive drugs and suppressed the utilization of non-winning drugs, indicated significant changes in drug utilization of policy-related drugs. In the long run, the policy will promote the overall improvement of drug quality at patient level. However, the NVBP policy was not associated with the decrease in the drug expenses and the healthcare burden of hypertension patients in the sample TCM hospital, which indicated deviation of policy implementation effects in the different medicine institutions.

The present study found that the utilization of bid-winning antihypertensive drugs remarkably increased in both outpatient and inpatient settings. Significant statistical increasing was observed in the ITS analysis of volume proportion for bid-winning drugs. As a consequence, the proportions of non-winning drug utilization dropped by 33.38% and 56.07% among patients treated in outpatient and inpatient settings, respectively. These results are consistent with those obtained in previous studies, the national policy steer changes in prescribing behaviour and contributes to the substitution use of bid-winning products (Chen et al., 2020; Yu, 2020; Wang et al., 2021b). The tread of changes in number of patients using bid-winning and non-winning consistent with the changes in volume proportion of the two categories (Supplementary Appendix SA1). At the patient level, the proportion of bid-winning prescriptions and the number of patients increased, meanwhile the proportion of hypertensive patients using bid-winng antihypertensive drugs also rose. The increased use of bid-winning drugs may be related to the significant price reduction that occurred after the implementation of the NVBP policy, and thus implied that the affordability of antihypertensive drugs increased significantly and released some unmet medication demands after drug price reduction (Wang et al., 2020b). Besides, to ensure the completion of the relevant policy assessment target, healthcare institutions have launched a series of incentives for doctors to prioritize recommending the bid-winning antihypertensive drugs (Liu et al., 2021; Lu et al., 2022b). As is well known, only generic drugs which have passed consistency evaluation are deemed eligible to participate in National Volume-based Procurement. With the continuous implementation of NVBP policy, the drug use of hypertension patients will increasingly focus on bid-winning drugs, which will significantly improve the overall quality of drug used at the population level (Hu, 2019; Liu, 2020). Meanwhile, the advance of policy will promote pharmaceutical enterprises to pay more attention to drug quality and innovation (Lu et al., 2022a). Research had found that the average price cut of the centralized Volume-Based drugs was about 52%, with a maximum decrease of 98%, which complies with the policy intention of improving drug accessibility.

It is widely recognized that the high expenditures of antihypertensive drugs is one of the important factors influenced the effective control of hypertension in China (Li et al., 2019). Wang et al.‘s found that the monthly drug expenditure of hypertension patients accounted for 16.38% of the total expenditure in Gansu Province, so family drug burden for those living with hypertension are the key challenges (Wang et al., 2020a). Prior to the implementation of the NVBP policy, only a very small amount of bid-winning drugs were obtained from enrolled hypertension patients in the HIS, with an average proportion of 0.06% in outpatient settings. After the policy implementation, the usage of the bid-winning antihypertensive drugs increased rapidly. The bid-winning drug expenditures averaging of 1.53 yuan per visit remained affordable although the significant level and trend change of bid-winning drug expenditures in outpatient setting after the policy were observed, and which indicated the policy effect of relieve the drug burden of hypertensive patients. However, some patients and doctors still had doubts about the safety and efficacy of the bid-winning drugs. It is necessary to explore more systematic approaches to boosting public confidence in bid-winning generic drugs and enhance physicians’ acceptability of them (Zhu et al., 2023).

Different changes were also found in the usage volume of non-VBP drugs in different healthcare settings. The ITS results showed that the immediate and long-term trends were stable, with no change in the trends among inpatients before and after the NVBP policy implementation. However, the usage proportion of non-VBP drugs increased by 5.34% after the NVBP policy was implemented, and with an upward trend observed among outpatients. The number of patients using non-VBP drugs both in outpatient and inpatient settings had no significant difference, with an observed upward tread before and after the policy (Supplementary Appendix SA1). Besides, the present study revealed that the average drug expenditures of hypertension patients in outpatient setting increased by 48.03% per visit, with non-VBP drug expenditure specifically increasing by a significant 123.10%. The expenditure of reduced-price drugs declined, but the use of non-VBP drugs increased significantly, which led to an increase in the drug expenditures and an increase in the medical burden of hypertension outpatients. The authors of this study have previously hypothesized that this increased in overall drug expenditure may be related to unreasonable prescription behaviors, such as increasing daily doses of antihypertensive drugs (Yang et al., 2021b). The increase in the proportional volume of non-VBP drugs was a so-called “side-effect” that is very commonly observed in relation to pharmaceutical policies (Kwon et al., 2019). Although the NVBP policy had promoted efficiency and eliminated the presence of gray profit in drug supply chain, service charges of medical institutions and physicians’ compensation systems did not change substantially (Fu et al., 2015). Therefore, compared with inpatient department, physicians operating in outpatient setting were more likely to change their prescription behavior by increasing the number of prescriptions to generate higher salaries. Compared to outpatients, inpatients might have a high degree of compliance with drug brands due to more severity of disease, and it is difficult for inpatients to change their medication habits to switch to bid-winning generic drugs (Wang et al., 2023). These considerations could also be some of the reasons for the differences in drug utilization between the outpatient and inpatient departments. Thus, further monitoring of clinical rational drug use and standardization of the prescription behavior of physicians are suggested after implementation of the policy.

In this study, it was found that the implementation of the NVBP policy improved the healthcare expenditures of hypertensive patients in inpatient setting. Notably, increments in expenditure were more prominent among consultations and other associated expenditures. Patients experienced an immediate and significant of 10,140.00 yuan in healthcare expenditure (*p = 0.024*), 1,490.40 yuan in consultation expenditure (*p = 0.008*) and 5,753.79 yuan in other associated expenditures (*p = 0.004*) after the implementation of NVBP policy. However, slope changes in all of the above expenditures were not obvious, indicating that the immediate effects of NVBP policy were noticeable, and the long-term trends were steady. Some studies have also reported that drug prices decreased after the implementation of centralized bidding system, whereas other non-drug expenditures continued to increase. Healthy institutions and physicians may provide patients with additional services, by overusing laboratory tests or unnecessary services to increase other non-drug expenditures such as service charges and operating costs (Li et al., 2021; Shen et al., 2021). These findings are generally in line with the results obtained in this study suggesting that price cuts alone cannot effectively lessen the burden on hypertensive patients. Drug price, drug volume, drug use structure and treatment mode affect the medical burden of patients. Therefore, it is recommended that relevant real-world investigations are necessary to generate a deeper understanding of the true effects of the NVBP policy. Moreover, medical supervision should be strengthened to reduce the increasing costs incurred by non-pharmaceutical companies.

In this research, using separate or unified policy initiation time yielded similar results. These reasons could be explained as follows. Firstly, the time interval between the first and last drugs affected by the policy is relatively short (approximately 1 year). Secondly, the policy could have a spill-over effect, causing all drugs to be affected due to substitution between anti-dyslipidemia products. Lastly, most of the drugs with a large volume were already affected in the first wave of NVBP, as the intended policy targets.

This study has several limitations. The primary limitation of this study is its reliance on data from a singular hospital, potentially constraining the generalizability of its findings. Notwithstanding this concern, the hospital in question ranks among the top three TCM institutions in Nanjing. This provides a measure of assurance that the results offer a representative evaluation of the NVBP policy’s impact on drug utilization and medical expenditure within hypertensive patients in pilot cities. Another limitation pertains to the quality of EHR data, where the authenticity of the results might be affected by non-standard data records. A specific concern emerged with missing outpatient data prior to 2019. To address this, we utilized prescription proportions across drug groups to probe volume changes in outpatient drug utilization due to the NVBP policy. This approach seeks to provide a clear representation of drug use changes pre and post NVBP policy enactment. Additionally, the nationwide implementation of the NVBP policy in public medical institutions complicates the establishment of an unaffected control group. This could introduce confounding variables affecting the research outcomes. As a countermeasure, we employed a single-group Interrupted Time Series (ITS), which could not exclude other factors that may affect the policy effects in this study. In spite of this, recognized as a robust quasi-experimental design, ITS is particularly effective and widely recognized method to access the policy effect when randomized controlled trials are infeasible. This method evaluates policy effects at a collective level (patient group) instead of an individual one. Additionally, the extended durations preceding and following the intervention ensure stability in estimates and diminished variability in the time series analysis. Thus, despite potential drawbacks, our results remain notably credible, grounding their foundation in the ITS analysis. Lastly, the study’s focus was primarily centered on drug utilization and expenditures of real-world hypertension patients. This means areas like the NVBP policy’s influence on the quality of bid-winning generic drugs, patient health outcomes, and patient adherence were not explored in depth. Future studies might delve into these aspects to comprehend the policy’s broader, long-term impacts on clinical outcomes.

## 5 Conclusion

The study addresses a significant gap in the literature, moving beyond the confines of purchase data to evaluate the real-world clinical implications of the NVBP policy within a Chinese medical institution. Post-policy intervention, a noticeable shift was observed in outpatient drug utilization, with an increase in the use of bid-winning and non-VBP drugs and a decrease in non-winning drugs. Interestingly, patterns in inpatient drug utilization differed from outpatient trends. Additionally, while the policy enhanced the preference for bid-winning drugs over non-winning counterparts, it coincided with an uptick in medical expenditures. This underlines the need for robust clinical use monitoring and integrated reforms across medical insurance, health institutions, and drug distribution systems to alleviate the financial strain on patients.

## Data Availability

The original contributions presented in the study are included in the article/Supplementary Material, further inquiries can be directed to the corresponding authors.
