# Supplementary material for: Impacts of national volume-based drug procurement policy on the utilization and costs of antihypertensive drugs in a Chinese medicine hospital: an interrupted time series analysis of 5138 patients
Source: Front Pharmacol. 2024 Feb 8;15:1302154. doi: 10.3389/fphar.2024.1302154 (PMC10881800; doi:10.3389/fphar.2024.1302154)
Supplement: Supplementary file 1 [file Table1.docx]

**The supplementary material**

Supplementary Table S1 General characteristics of bid-winning and non-winning antihypertensive drugs

| **Generic name** | **Enterprises** | **Specifications Dosage/package** | **Formulation** | **Bid-winning or non-winning** | **Implementation time** |
| --- | --- | --- | --- | --- | --- |
| Amlodipine | Sinopharm Ronshyn Pharmaceuticals Co.,Ltd | 5mg*14 | Tablet | Yes | 2020.1.1 |
|  | Pfizer Pharmaceuticals Ltd (Da lian)^△^ | 5mg*7 | Tablet | No |  |
| Bisoprolol | Chengdu Easton Bio Pharmaceuticals Co.,Ltd | 2.5mg*18 | Tablet | Yes | 2020.4.27 |
|  | Merck Pharmaceuticals GmbH, Germany^△^ | 5mg*10 | Tablet | No |  |
| Captopril | Shiyao Group Ouyi Pharmaceutical Co.,Ltd | 25mg*100 | Tablet | Yes | 2020.11.1 |
|  | SPH Changzhou Pharmaceutical Co.,Ltd | 25mg*100 | Tablet | No |  |
|  | SPH Changzhou Pharmaceutical Co.,Ltd | 25mg*100 | Tablet | No |  |
|  | Sino-AmericanShanghai Squibb Pharmaceuticals Co.,Ltd | 12.5mg*20 | Tablet | No |  |
| Fosinopril | Sino-American Shanghai Squibb Pharmaceuticals Ltd^△^ | 10mg*14 | Tablet | Yes | 2020.1.1 |
| Irbesartan | Zhejiang Huahai Pharmaceutical Co.,Ltd | 75mg*28 | Tablet | Yes | 2020.1.1 |
|  | Sanofi (Hang zhou) Pharmaceutical Co., Ltd ^△^ | 150mg*7 | Tablet | No |  |
|  | Jiangsu Hengrui Pharmaceuticals Co.,Ltd | 150mg*14 | Tablet | No |  |
| Irbesartan hydrochlorothiazide | Zhejiang Huahai Pharmaceutical Co.,Ltd | (150mg+12.5mg)*28 | Tablet | Yes | 2020.1.1 |
|  | Sanofi (Hang zhou) Pharmaceutical Co.,Ltd ^△^ | (150mg+12.5mg)*7 | Tablet | No |  |
| L-Amlodipine | Jilin Tianfeng Pharmaceutical Co., Ltd | 2.5mg*14 | Tablet | No |  |
|  | Shi Huida Pharmaceutical Group (Ji lin) Co.,Ltd | 2.5mg*14 | Tablet | No |  |
| Losartan | Zhejiang Huahai Pharmaceutical Co.,Ltd. | 50mg*7 | Tablet | Yes | 2020.1.1 |
|  | Beijing Wansheng Pharmaceutical Co.,Ltd | 50mg*7 | Capsule | No |  |
|  | Fuyuan Pharmaceutical Co., Ltd | 50mg*14 | Capsule | No |  |
|  | Hangzhou MSD Pharmaceutical Co.,Ltd^△^ | 100mg*7 | Tablet | No |  |
|  | Hangzhou MSD Pharmaceutical Co.,Ltd^△^ | 50mg*7 | Tablet | No |  |
|  | Hangzhou MSD Pharmaceutical Co.,Ltd^△^ | 100mg*7 | Tablet | No |  |
| Olmesartan medoxomil | Shenzhen Salubris Pharmaceuticals Co.,Ltd | 20mg*14 | Tablet | Yes |  |
|  | Daiichi Sankyo Pharmaceutical (Shang hai) Co.,Ltd | 20mg*7 | Tablet | No |  |
|  | Zhengda Tianqing Pharmaceutical Group Co.,Ltd | 20mg*7 | Tablet | No |  |
| Valsartan | Tianda Pharmaceutical Zhuhai Co.,Ltd | 80mg*7 | Capsule | Yes | 2020.1.1 |
|  | Beijing Novartis Pharma Ltd^△^ | 80mg*7 | Tablet | No |  |
|  | Changzhou Siyao Pharmaceutical Co.,Ltd | 40mg*14 | Capsule | No |  |
| Valsartan amlodipine | Jiangsu Hengrui Pharmaceutical Co.,Ltd | (80mg+5mg)*7 | Tablet | Yes | 2020.4.27 |
|  | Beijing Novartis Pharma Ltd^△^ | (80mg+5mg)*7 | Tablet | No |  |
| Valsartan hydrochlorothiazide | China Resources SAIKE Pharmaceutical Co.,Ltd | (80mg+12.5mg)*14 | Tablet | Yes | 2021.4.27 |
|  | Novartis Pharmaceuticals AG, Switzerland^△^ | 80mg*7 | Tablet | No |  |

Note. ^△^ original drug

Supplementary Table S2 ITS results of volume proportion of bid-winning, non-winning and non-VBP antihypertensive drugs in outpatients and inpatients when unifying the execution time to January 1, 2020

| **Categories** | **Outpatients** | | **Inpatients** | |
| --- | --- | --- | --- | --- |
|  | **Coef.** | **95 % CI** | **Coef.** | **95 % CI** |
| **Bid-winning** |  |  |  |  |
| Level change,β2 | 9.56 | (6.86, 12.26)*** | 6.38 | (5.11, 7.65)*** |
| Trend change,β3 | 0.13 | (-0.22, 0.49) | 0.37 | (0.24, 0.49)*** |
| Durbin-Watson,d | 2.05 |  | 1.76 |  |
| **Non-winning** |  |  |  |  |
| Level change,β2 | -15.84 | (-19.19, -12.50)*** | -11.33 | (-19.89, -2.76)* |
| Trend change,β3 | -0.91 | (-1.33, -0.49)*** | -0.33 | (-1.17, 0.55) |
| Durbin-Watson,d | 2.06 |  | 1.89 |  |
| **Non-VBP** |  |  |  |  |
| Level change,β2 | 5.30 | (2.36, 8.25)** | 7.82 | (-0.21, 15.85) |
| Trend change,β3 | 0.76 | (0.38, 1.13)*** | -0.01 | (-0.83, 0.76) |
| Durbin-Watson,d | 2.00 |  | 1.98 |  |

Abbreviations: Coef., coefficient; SE, standard error; CI, confidence interval.

*p < 0.05, **p < 0.01, ***p < 0.001

Supplementary Table S3 ITS results of absolute prescription volume of bid-winning, non-winning and non-VBP antihypertensive drugs in outpatients and inpatients

| **Categories** | **Outpatients** | | **Inpatients** | |
| --- | --- | --- | --- | --- |
|  | **Coef.** | **95 % CI** | **Coef.** | **95 % CI** |
| **Bid-winning** |  |  |  |  |
| Level change,β2 | 56.76 | (42.12, 71.40)*** | 44.89 | (32.95, -8.36)*** |
| Trend change,β3 | 2.88 | (1.04, 4.72) | 3.42 | (2.24, -10.56)*** |
| Durbin-Watson,d | 2.08 |  | 1.89 |  |
| **Non-winning** |  |  |  |  |
| Level change,β2 | -281.31 | (-363.30, -199.31)*** | -116.58 | (-167.33, 34.72)** |
| Trend change,β3 | -13.94 | (-24.87, -3.01)** | -3.94 | (-11.97, 5.98) |
| Durbin-Watson,d | 1.85 |  | 1.99 |  |
| **Non-VBP** |  |  |  |  |
| Level change,β2 | -141.45 | (-228.08, -54.83)** | -143.10 | (-319.38, 93.06) |
| Trend change,β3 | 2.49 | (-9.02, 14.00)** | 5.99 | (-16.64, 10.53) |
| Durbin-Watson,d | 1.92 |  | 2.22 |  |

Abbreviations: Coef., coefficient; SE, standard error; CI, confidence interval.

*p < 0.05, **p < 0.01, ***p < 0.001

Appendix SA1 ITS results of the proportion of patients number using bid-winning, non-winning and non-VBP antihypertensive drugs in outpatient and inpatient setting

| **Categories** | **Outpatients** | | **Inpatients** | |
| --- | --- | --- | --- | --- |
|  | **Coef.** | **95 % CI** | **Coef.** | **95 % CI** |
| **Bid-winning** |  |  |  |  |
| Level change,β2 | 14.04 | (11.76, 16.31)*** | 18.42 | (15.73, 21.10)*** |
| Trend change,β3 | 0.24 | (-0.04, 0.52) | 0.66 | (0.39, 0.92)*** |
| **Non-winning** |  |  |  |  |
| Level change,β2 | -18.57 | (-22.06, -15.09)*** | -18.02 | (-25.25, -10.80)*** |
| Trend change,β3 | -0.63 | (-1.07, -0.19)** | -0.81 | (-1.52, -0.10)* |
| **Non-VBP** |  |  |  |  |
| Level change,β2 | 3.55 | (-0.15, 7.25) | 4.69 | (-4.53, 13.91) |
| Trend change,β3 | 0.22 | (-9.02, 14.00) | -0.17 | (-1.09, 0.76) |

Abbreviations: Coef., coefficient; SE, standard error; CI, confidence interval.

*p < 0.05, **p < 0.01, ***p < 0.001
